# White Matter Matters: A Magnetic Resonance Imaging Study with Clinical Correlates in Primary Brain Calcification

**DOI:** 10.1002/mds.70249

**Published:** 2026-02-21

**Authors:** Giovanni Librizzi, Giulia Bonato, Matilde Corazza, Irene Guerra, Francesca Pistonesi, Cinzia Bertolin, Leonardo Salviati, Angelo Antonini, Renzo Manara, Miryam Carecchio

**Affiliations:** ^1^ Neuroradiology Unit, DIMED, University of Padova Padova Italy; ^2^ Padova Neuroscience Center (PNC) University of Padova Padova Italy; ^3^ Neurodegenerative Disease Unit, Department of Neuroscience University of Padova Padova Italy; ^4^ Clinical Genetics Unit, Azienda Ospedale Università Padova Padova Italy; ^5^ Clinical Genetics Unit, Department of Women and Children's Health University of Padova Padova Italy; ^6^ IRCCS San Camillo Venice Italy

**Keywords:** calcification, cognition, Fahr, MRI, PBC, white matter

## Abstract

**Background:**

Primary brain calcification (PBC) is a genetic disease featuring movement disorders, cognitive impairment, and/or psychiatric symptoms. Computed tomography (CT) scan identifies brain calcification but poorly correlates with patients' clinical phenotype; the role of magnetic resonance imaging (MRI) is yet undefined.

**Objective:**

Describing white matter (WM) changes and clinical correlates in PBC.

**Methods:**

Fifty PBC patients and 50 age‐matched controls underwent 3 T brain MRI. Patients also underwent brain CT scan, genetic analysis, and clinical and neuropsychological evaluation. Two patterns of supratentorial WM changes were observed: a leukodystrophic band‐like and a scattered vascular‐like one that were characterized based on anatomical location and severity. Cerebellar WM alterations were also described. Comparison tests and multivariate analysis were applied.

**Results:**

WM abnormalities were found in 41/50 patients and 32/50 controls. Supratentorial band‐like leukopathy was observed in 21/25 patients with centrum semiovale calcifications, involved the deep/periventricular regions with intermediate sparing (19/21), was frequently severe (15/21), diffuse (10/21) or with anterior prevalence (10/21), and mostly associated with *MYORG* (8/9) and *PDGFB*/*PDGFRB* (5/8) mutations. The prevalence and severity of vascular‐like scattered abnormalities did not differ between patients and controls. Cerebellar leukopathy was present in 16/50 patients, being severe in 11/16, frequently observed in *MYORG* patients (9/10), and associated with band‐like supratentorial leukopathy (16/16) and cerebellar hemispheric calcifications. WM involvement correlated with cognitive impairment, parkinsonism, psychiatric disturbances (band‐like pattern, *P* = 0.02), and cerebellar symptoms (cerebellar leukopathy, *P* = 0.002).

**Conclusions:**

WM alterations are frequent in PBC and may be an expression of leukodystrophy‐like tissue changes, being a potential imaging biomarker of cognitive impairment, parkinsonism severity, and cerebellar dysfunction. © 2026 The Author(s). *Movement Disorders* published by Wiley Periodicals LLC on behalf of International Parkinson and Movement Disorder Society.

Primary brain calcification (PBC), formerly known as Fahr's disease, is a genetic neurodegenerative disorder of adulthood characterized by calcium deposits in the basal ganglia (BG), with additional supra‐ and infratentorial anatomical regions possibly involved.^1^, ^2^, ^3^ Marked differences in disease clinical penetrance, symptoms, and onset^2^, ^4^ are known. About two‐thirds of PBC patients feature motor impairment (especially parkinsonism), cognitive decline, or psychiatric disorders, while others remain lifelong asymptomatic.^4^, ^5^ Seven causative genes are known, with autosomal dominant (*SLC20A2*, *PDGFB*, *PDGFRB, XPR1*) and recessive (*MYORG*, *JAM2, NAA60*) inheritance^6^, ^7^, ^8^ but the mutated gene remains unknown in about half of patients.

Computed tomography (CT) has been traditionally considered the gold standard technique for PBC diagnosis, and the total calcification score (TCS) was proposed to assess semiquantitatively the severity of cerebral calcifications.^9^ However, TCS only partially correlates with type and severity of patients' clinical manifestations,^2^, ^4^, ^10^ and the heterogeneity of disease course and prognosis remain largely unexplained.

Brain magnetic resonance imaging (MRI) is more sensitive for detecting disease‐related tissue changes, often complementing CT, yet it has been less commonly used in PBC because of its lower sensitivity for detecting calcifications. In a previous MRI study on PBC,^11^ a relationship between cognitive impairment and cerebral white matter (WM) changes was observed, and it was suggested that WM changes could be an imaging biomarker of clinical involvement. However, the study focused on supratentorial regions only, WM changes were poorly characterized, and genetic data were not available. Subsequent case reports highlighted extensive WM involvement akin to leukodystrophy mostly in *PDGFB* mutation carriers,^12^, ^13^, ^14^, ^15^, ^16^ but also in other causative genes (*SCL20A2*, *XPR1*) in patients with heterogeneous clinical severity.^16^, ^17^, ^18^ However, alterations of cerebral WM and its clinical relevance in PFBC have never been systematically investigated, despite of an increasingly recognized functional role of WM in healthy and diseased brain.^19^, ^20^, ^21^

The present MRI study aimed to characterize the supra‐ and infratentorial WM changes in a cohort of PBC patients, their relationship with disease course and genetic etiology, and their contribution to determining patients' clinical phenotype.

## 1 Methods

This was a case–control, cross‐sectional MRI and clinical study on a PBC cohort followed at the Neurology Department of Padua University that included 87 patients with a TCS above the threshold for age group (> 0 for age < 40 years, > 4 for age between 40 and 60 years, > 5 for age > 60 years),^9^ in which secondary causes of brain calcification were appropriately excluded.^5^, ^22^

Fifty‐two patients underwent a brain MRI from November 2023 to April 2025; the remaining 35 subjects were not included because of MRI contraindication, no compliance, intercurrent death, or severe disability. Two MRI scans were excluded due to comorbidities interfering with imaging evaluation (normal pressure hydrocephalus and prior radiotherapy, respectively), leading to a final cohort of 50 patients.

PBC participants and non‐participants' features were comparable, except for genetic diagnosis (Supplementary Table S1).

Fifty gender‐ and age‐matched controls enrolled on a voluntary basis, without known neurological diseases and no statistically significant difference in the rate of cardiovascular risk factors, underwent an identical MRI protocol. Imaging findings and/or history of head trauma, brain tumors, inflammatory, and developmental disorders were considered exclusion criteria.

All patients underwent genetic testing: Next generation sequencing (NGS) Illumina NextSeq platform, Agilent SureSelect kit, custom‐made panel including all known calcification‐related genes (list of genes available from the authors on request); American College of Medical Genetics and Genomics‐Association for Molecular Pathology (ACMG‐AMP) guidelines were followed for interpretation.^22^, ^23^ Thirty‐four patients were previously included in two publications from our group,^5^, ^22^ of which 10 were genetically undetermined, 9 *SLC20A2*, 8 *MYORG*, 4 *PDGFB*/*PDGFRB*, 1 *XPR1*, and 2 *JAM2* with single variant.

All brain MRI scans were performed using a Philips Ingenia 3.0 T (Best, The Netherlands) MRI scanner with a 32‐channel head coil. The MRI protocol included the following sequences: three‐dimensional (3D) fluid‐attenuated inversion recovery (FLAIR; TE: 360 ms, TR: 8000 ms, TI: 2400 ms; acquisition voxel size: 1.12 mm; reconstructed slice thickness:3 mm), 2D‐diffusion weighted imaging (DWI; TR/TE: 4108/105 ms; *b*‐value: 1500s^2^/m; slice thickness: 4 mm; slice gap: 1 mm; averages: 2).

The scans were evaluated by one senior and one junior neuroradiologist; discordant assessment was resolved by consensus. Each patient's TCS was calculated on CT scans performed in the same period of time; involvement of cerebellar cortex was also considered (present/absent). WM changes were assessed by FLAIR and DWI sequences (including apparent diffusion coefficient [ADC] maps). Supratentorial WM (centrum semiovale [CSO], corona radiata, internal capsule) and cerebellar medullary body/corpora (MDB) were considered for evaluation.

**FIG. 1:**
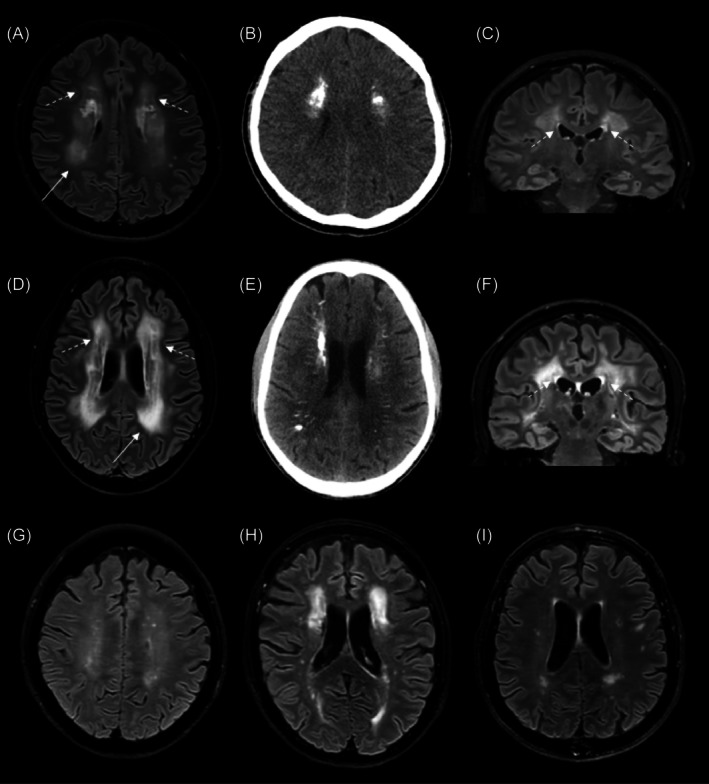
White matter involvement in primary brain calcification. Brain magnetic resonance imaging (MRI) scans in a 46‐year‐old male *MYORG* mutation carrier and in a 31‐year‐old male *PDGFB* mutation carrier showing severe diffuse centrum semiovale band‐like leukopathy (A, D, full arrows) extending beyond calcifications visible on computed tomography (CT) (B, E). The signal alteration involves both deep and periventricular white matter, but intermediate fibers are spared (C, F, dashed arrows). Brain MRI in an 81‐year‐old female *PDGFB* mutation carrier showing mild diffuse centrum semiovale band‐like leukopathy coexisting with punctate scattered lesions (G). Brain MRI in a 72‐year‐old female *PDGFB* mutation carrier showing severe anterior centrum semiovale band‐like leukopathy coexisting with punctate scattered lesions (H). Brain MRI in a 78‐year‐old male showing multiple initially confluent scattered vascular‐like Fazekas score 2 lesions (I).

Supratentorial WM alterations were classified as: (1) absent, (2) vascular‐like scattered lesions of the CSO, or (3) leukodystrophic band‐like hyperintensity (WM intertwined with thinner antero‐posteriorly oriented layers of normal appearing WM). In addition, we described: regional pattern of involvement (global or mainly anterior/posterior, symmetric or asymmetric), anatomical location (periventricular [<1 cm from ependyma], deep, and/or juxtacortical [<0.5 cm from cortex]), and severity (mild or severe) of WM alterations (Fig. 1).

After excluding patients with band‐like signal alterations, the Fazekas scale^24^ was used to compare CSO WM alterations in patients and controls. Territorial, lacunar strokes, and recent ischemic lesions on DWI sequences were recorded.

**FIG. 2:**
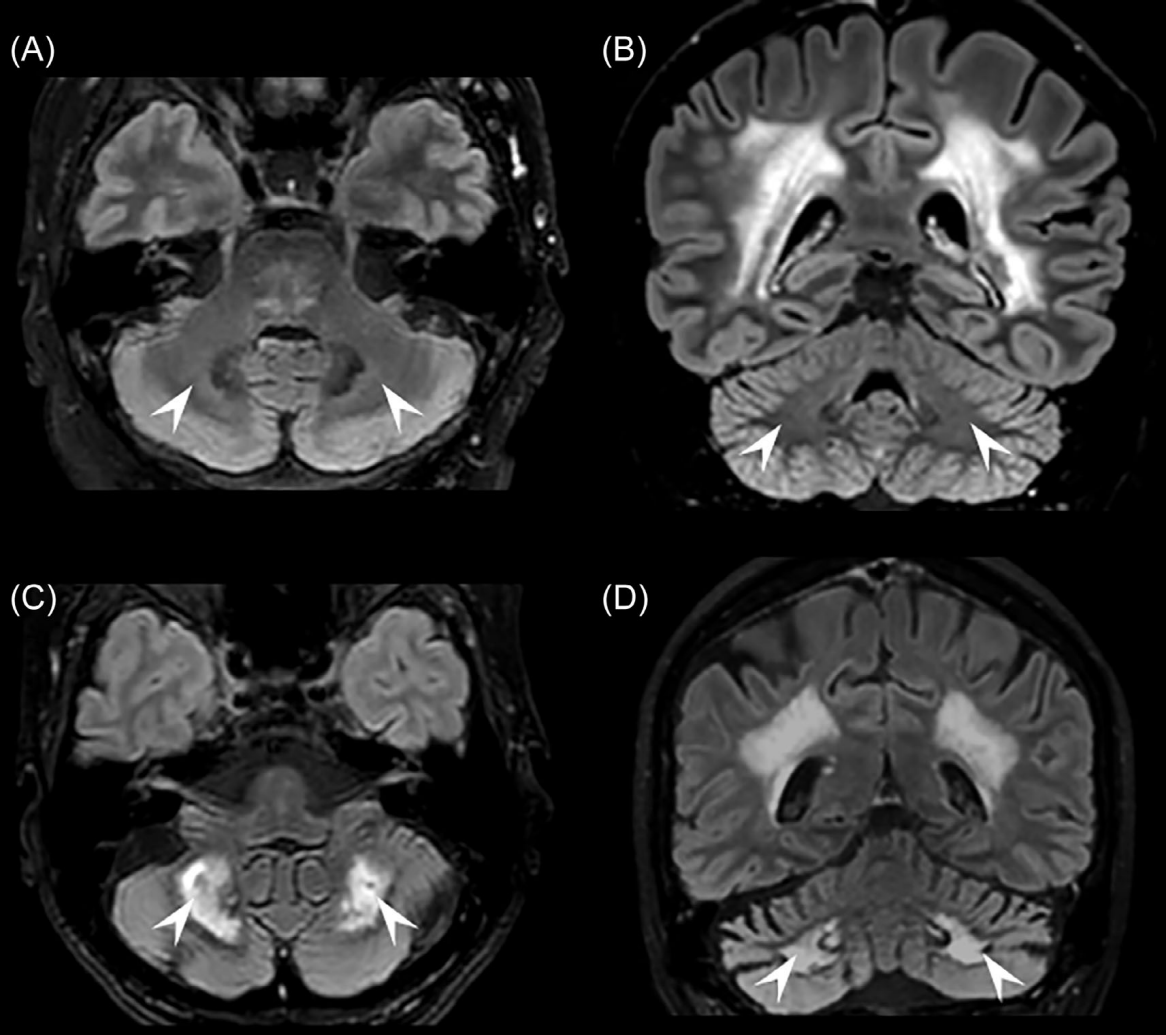
Cerebellar brain magnetic resonance imaging (MRI), fluid‐attenuated inversion recovery (FLAIR) images: (A) axial image in a 68‐year‐old *SLC20A2* female showing mild cerebellar hemispheric leukopathy; (B) coronal image in a 31‐year‐old *MYORG* male (A, B) with mild cerebellar hemispheric leukopathy; severe supratentorial band‐like leukopathy was also recognizable; (C, D) axial and coronal images in a 71‐year‐old *MYORG* female revealing severe cerebellar hemispheric leukopathy with subcortical white matter sparing; severe supratentorial band‐like leukopathy is also evident in (D).

Cerebellar WM involvement was rated as absent, mild, or severe; symmetry of involvement was also considered (Fig. 2). The relationship between WM changes and severity of calcifications expressed as TCS was examined.

A complete neurological and neuropsychological assessment was performed to define patients' motor and cognitive status, as previously reported.^5^ Montreal Cognitive Assessment (MoCA) was used to evaluate global cognitive functions and affected cognition areas; subjects with a MoCA score < 26 or subjective complaints were evaluated with second‐level tests further exploring language, memory, attention, and executive and visuospatial functions in order to rule out a possible mild cognitive impairment (MCI) (complete lists of tests and normative values are available from the authors on request); alterations in each domain was scored as present/absent.^25^, ^26^ MoCA scores were corrected for age and education level. One patient was excluded from cognitive tests quantitative analysis due to a language barrier; however, no cognitive complaints were reported upon history collection.

Anxiety and depression, the most frequent neuropsychiatric manifestations of PBC, were assessed through clinical history collection and dedicated scales (Beck Depression Inventory‐II [BDI‐II], State–Trait Anxiety Inventory [STAI] Y‐1/Y‐2) and scored as present/absent.^5^

Motor symptoms (parkinsonism and cerebellar signs) were categorized as present/absent and the Movement Disorder Society Unified Parkinson's Disease Rating Scale‐Part III (MDS‐UPDRS‐III) was used to score parkinsonism.^27^ Group comparison was conducted with chi‐squared or Fisher test for qualitative data, Mann–Whitney for ordinal data, and Kruskal–Wallis‐test or Student's *t*‐test for quantitative data. Multivariate analysis of the association of band‐like supratentorial and cerebellar MDB leukopathy with clinical and radiological variables was conducted using a bias‐corrected generalized linear model. Clinical predictors included age, gender, and genetics. Radiological predictors included lenticular, caudate, and supratentorial subcortical WM TCS subscores for band‐like supratentorial leukopathy; cerebellar hemispheric TCS subscore, and the presence of cerebellar cortical calcifications for cerebellar leukopathy. Due to the expected relative symmetry in left and right calcification severity, the sum of left and right TCS subscores was used in the model.

The study was conducted in accordance with the Declaration of Helsinki and ethics guidelines from the home institution and approved by the local ethics committee (Protocol number: 528n/AO/24). All participants signed an informed consent form for data collection and study procedures.

## 2 Results

Fifty patients (mean age: 56.5 ± 12.6 years; 26 females) from 48 different families were included; the main clinical and demographic data and TCS scores are reported in Table 1.

**TABLE 1:** Clinical and demographic data and total calcification scores of primary brain calcification patients

Parameter	Cohort description
Patients, n (%)	50 (100%)
Females, n (%)	26 (52%)
Age at MRI (years), mean ± SD	56.5 ± 12.6
Disease duration (years), mean ± SD	10.6 ± 9.9
TCS, mean ± SD	23.1 ± 18.9
Clinical manifestations, n (%)	
Movement disorders	28 (56%)
Parkinsonism	19 (38%)
Cerebellar symptoms	11 (22%)
Dysarthria	12 (24%)
Tremor	16 (32%)
Dystonia	8 (16%)
Cognitive decline	21 (42%)
MCI	20 (40%)
Dementia	1 (2%)
Neuropsychiatric symptoms (anxiety/depression)	36 (72%)
Asymptomatic	10 (20%)
Genetic variants, n (%)	34/50 (68%)
SLC20A2	12 (24%)
MYORG	9 (18%)
PDGFB	7 (14%)
PDGFRB	1 (2%)
XPR1	1 (2%)
HetAR	4 (8%)
Negative test, n (%)	16/50 (32%)

Abbreviations: MRI, magnetic resonance imaging; SD, standard deviation; TCS, total calcification score; MCI, mild cognitive impairment; HetAR, single heterozygous variants in autosomal recessive genes.

Variants in PBC‐related genes were found in 34/50 subjects; in 30 cases, variants were classified as pathogenic or likely pathogenic also based on segregation analysis and clinical–radiological phenotype. Four symptomatic patients had a single ACMG‐AMP class 3 variant in a recessive gene (3 *JAM2*, 1 *MYORG*).^5^ These patients likely carry a second, possibly intronic DNA variant in the same recessive genes, not individuated by the NGS technique used, not allowing a fully appropriate genetic classification in the positive/negative group. For this reason, they were excluded from statistical analysis.

Overall, supratentorial WM abnormalities were mainly hyperintense on FLAIR sequences with increased ADC values and were observed in 41/50 patients (82%); none showed recent ischemic lesions on DWI sequences. Band‐like supratentorial leukopathy was found in 21/50 (42%) patients, scattered CSO lesions in 32/50 (64%), with 12 patients showing both band‐like and scattered supratentorial leukopathy.

Some 10/21 (47.6%) patients with band‐like supratentorial leukopathy had a diffuse hemispheric involvement with no detectable anterior/posterior gradient, 10/21 (47.6%) had an anterior prevalence, and only 1/21 (4.8%) showed a posterior prevalence. In all subjects, an involvement of deep supratentorial WM was observed, with spreading to the periventricular regions in 19/21 (90.5%) subjects and, in the most severe cases, to the juxtacortical regions (9/21; 42.9%), even though the U‐fibers were spared in all cases. The band‐like involvement was classified as severe in 15/21 (71.4%) and mild in 6/21 (28.6%) cases, and in most patients (19/21; 90.5%) there was a deep and periventricular alteration with faint intermediate sparing (Fig. 1). Age did not differ between patients with mild or severe band‐like involvement (range 51–72 vs. 31–72 years, mean 62.3 ± 8.2 vs. 56.4 ± 12.9 years, *P* = 0.32). No association between the anatomical pattern (diffuse, anterior, or posterior) and the severity of leukopathy was found (*P* = 0.33).

Band‐like involvement was more common in patients with a definite genetic diagnosis (16/30, 53.3%) than in those without (2/16, 12.5%, *P* = 0.01), especially in *MYORG* (9/10 subjects, 90%) and *PDGFB/PDGFRB* (5/8 subjects, 62.5%) mutation carriers, regardless of their age (range 45–68 years and 30–71 years, respectively), whereas it was less frequent among other genetic subtypes (3/12, 25% in *SLC20A2*).

The presence of supratentorial band‐like leukopathy was significantly associated with a higher TCS (37.6 ± 18.6 vs. 12.6 ± 10.2, *P* < 0.001) and with higher severity of calcification of the CSO WM (*P* < 0.001), lenticular (*P* = 0.01), and caudate (*P* = 0.003) nuclei expressed as TCS subscores. On multivariate analysis, only CSO WM calcifications were significantly associated with band‐like changes (*P* = 0.002): all patients with band‐like WM changes also had CSO calcifications, and 21/25 (84%) patients with CSO calcifications had a WM band‐like involvement.

Fazekas score was not calculated in patients with band‐like supratentorial WM abnormalities, and these subjects were not considered for group comparison, as they would have intrinsically scored 3 due to the leukodystrophy‐like appearance. Fazekas scores of 0, 1, 2, and 3 were observed in 9, 16, 3 and 1 patients, respectively, and in 18, 27, 3, and 2 controls, with no difference between these groups (*P* = 0.89). Fazekas score increased with patients' age (*P* = 0.02). A definite genetic diagnosis was not associated with a Fazekas score > 0 (13/20 vs 3/9, *P* = 0.226). In addition, TCS and rate of cerebral infarction were higher among patients with higher Fazekas scores, but the association was not significant (*P* = 0.10 and *P* = 0.38, respectively).

Cerebellar WM alterations on FLAIR sequences were found in 16/50 (32%) PBC patients and in no controls, being severe in 12/50 (24%), mild in 4/50 (8%), and more frequent in cases with a genetic diagnosis (13/30 vs. 1/16, *P* = 0.016). Severe cerebellar WM involvement was almost universal in *MYORG* mutation carriers, being present in 8/9 patients (*P* < 0.001) compared with 3/8 *PDGFB*/*PDGFRB* and 2/12 *SLC20A2* mutation carriers. Patients with cerebellar WM alterations had overall a higher TCS than those without (44.4 ± 15.6 vs. 13.1 ± 9.7, *P* < 0.001), whereas no significant TCS difference was found between severe and mild WM involvement (45.5 ± 15.4 vs. 41.3 ± 18.2). Cerebellar leukopathy was more frequent in patients with cerebellar cortical calcifications (15/16 vs 6/34, *P* < 0.001) and in those with a higher severity of hemispheric (2.38 ± 1.63 vs 0.06 ± 0.24, *P* < 0.001) and vermian (4.56 ± 0.63 vs 1.03 ± 1.27, *P* < 0.001) calcifications calculated with TCS subscores. On multivariate analysis, only cerebellar hemispheric calcification score was significantly associated with leukopathy (*P* = 0.04).

Cerebellar leukopathy was also associated with band‐like supratentorial leukopathy (*P* < 0.001) while, among the latter, 15/21 had cerebellar leukopathy (Table 2).

**TABLE 2:** Association between supratentorial band‐like and cerebellar leukopathy severity

		Supratentorial band‐like involvement		
		Severe	Mild	Absent
Medullary corpora involvement	Severe	11	1	0
	Mild	2	1	1
	Absent	1	4	29

From a clinical standpoint, cognitive impairment of any degree was associated with supratentorial WM involvement, being present in 35% and 62% of patients with scattered or band‐like WM involvement, respectively, whereas it was uncommon (11%) among patients without WM changes (*P* = 0.02). Adjusted MoCA scores also correlated with the presence of leukopathy (no changes vs. scattered vs. band‐like pattern: 25.1 ± 4.0 vs. 24.2 ± 3.0 vs 21.3 ± 4.0, *P* = 0.01). In the band‐like subgroup, the severity of WM changes, but not the diffuse/anterior pattern, was associated with lower MoCA scores, though not reaching statistical significance (Table 3). Among patients with no band‐like involvement, cognitive performances did not differ according to the presence or absence of scattered WM alterations (*P* = 0.3); there was no significant association between the Fazekas and MoCA scores, though there was a tendency to worse cognitive performance and MCI frequency in subjects with a higher Fazekas score.

**TABLE 3:** Supratentorial white matter involvement in primary brain calcification patients according to demographics, total calcification score, and clinical findings

WM involvement		Sample size	Age (years)	Female sex	TCS	MCI/dementia	MoCA	Motor impairment	Cerebellar deficits	Psychiatric symptoms	MDS‐UPDRS‐III
Non‐band‐like		29	55.2 ± 13.0	14 (48%)	12.6 ± 10.5	8 (27%)	24.5 ± 3.3	14 (48%)	2 (7%)	17 (58%)	4.2 ± 6.4
None	Fazekas 0	9	48.2 ± 11.8	4 (44%)	9.4 ± 7.5	1 (11%)	25.1 ± 4.0	4 (44%)	1 (11%)	3 (33%)	3.9 ± 6.5
Scattered	Fazekas 1–3	20	58.4 ± 12.5	10 (50%)	14.0 ± 11.2	7 (35%)	24.2 ± 3.0	10 (50%)	1 (5%)	14 (70%)	4.4 ± 6.6
	Fazekas 1	16	54.7 ± 10.5	8 (50%)	11.3 ± 6.5	5 (31%)	24.4. ± 2.8	7 (44%)	0 (0%)	13 (81%)	2.1 ± 3.1
	Fazekas 2	3	70.7 ± 8.1	1 (33%)	31.0 ± 18.1	2 (67%)	21.7. ± 2.4	2 (67%	0 (0%)	1 (33%)	12.7 ± 11.7
	Fazekas 3	1	81	1 (100%)	6	0 (0%)	28.4	1 (100%)	1 (100%)	0 (0%)	15
Band‐like	All	21	58.1 ± 11.8	12 (57%)	37.5 ± 18.7	13 (62%)	21.3 ± 4.0	14 (67%)	9 (43%)	19 (90%)	12.1 ± 15.4
	Mild	6	62.3 ± 8.2	5 (83%)	18.5 ± 10.9	3 (50%)	23.1 ± 3.7	2 (33%)	1 (17%)	5 (83%)	5.2 ± 9.8
	Severe	15	56.4 ± 12.8	7 (47%)	45.1 ± 15.5	10 (66%)	20.6 ± 4.1	12 (80%)	8 (53%)	14 (93%)	14.9 ± 16.6
	Anterior/ posterior	11	59.4 ± 10.8	8 (73%)	39.1 ± 18.6	7 (64%)	21.2 ± 5.0	6 (55%)	3 (27%)	11 (100%)	10.6 ± 16.9
	Diffuse	10	56.7 ± 13.3	4 (40%)	35.8 ± 19.7	6 (60%)	21.4 ± 2.9	8 (80%)	6 (60%)	8 (80%)	13.8 ± 14.2

Abbreviations: WM, white matter; TCS, total calcification score; MCI, mild cognitive impairment; MoCA, Montreal Cognitive Assessment; MDS‐UPDRS‐III, Movement Disorder Society Unified Parkinson's Disease Rating Scale‐Part III.

In addition, cognitive impairment was more common in patients with WM alterations in the cerebellum (11/16 vs. 10/34, *P* = 0.01; mean adjusted MoCA score 20.9 ± 4 vs. 24.1 ± 3.2, *P* = 0.005) and it was associated with a higher total TCS and WM TCS subscores (*P* < 0.01).

On multivariate analysis, a band‐like pattern was a predictor of memory domain involvement (*P* = 0.04) on neuropsychological assessment, whereas the TCS was a predictor of attention involvement (*P* < 0.01).

In terms of motor involvement, cerebellar signs were more common in patients with WM alterations in the cerebellar MDB (8/16 vs. 3/34, *P* = 0.002). The presence of non‐cerebellar motor symptoms did not correlate with WM changes, being detected in 14/21 (67%) patients with band‐like and 10/20 (50%) with scattered only supratentorial WM changes, in 11/16 (69%) with cerebellar WM changes but also in 4/9 (44%) patients without WM involvement. However, MDS‐UPDRS‐III score was higher in patients with WM band‐like involvement (12.1 ± 15.4 vs. 4.2 ± 6.4, *P* = 0.02), regardless of a diffuse or anterior/posterior involvement but with a trend of association with a severe involvement (severe 14.9 ± 16.6 vs. mild 5.2 ± 9.8, *P* = 0.08). MDS‐UPDRS‐III score also tended to be higher in patients with a greater scattered lesion burden, but without statistical significance (Fazekas score 0–1 2.72 ± 4.6 vs. Fazekas score 2–3 13.32 ± 9.6, *P* = 0.053).

A higher frequency of anxiety and depression was observed in patients with band‐like involvement (19/21 vs. 17/29, *P* = 0.02). Neuropsychiatric disturbances were more frequent in subjects with cerebellar leukopathy, though the difference did not reach the significance (13/16 vs. 23/34, *P* = 0.1).

## 3 Discussion

In this MRI study we investigated WM changes in a cohort of PBC patients, showing their heterogeneity in terms of severity, anatomical location, and their relationship with genetics and clinical manifestations.

The first MRI report of supratentorial WM changes in PBC in 1985^28^ showed a diffuse and symmetric involvement of CSO. Subsequently, the involvement of supratentorial WM was associated with the presence of cognitive deficits.^11^ Since then, more than 20 articles reporting on supratentorial WM appearance in PBC have been published (Supplementary Table S2), mainly single case reports with an ancillary WM assessment or limited descriptions of MRI images. The present study distinguished two MRI patterns of supratentorial WM changes: (1) a regionally homogeneous and symmetric band‐like fronto‐parietal alteration with U‐fiber sparing resembling a leukodystrophy and (2) a punctate, variably prominent vascular‐like involvement. Such changes seem to be independent of each other and might coexist as observed in about 25% of patients. Whenever this distinction was possible on available imaging, the pattern of leukopathy in previously reported cases was mostly band‐like (22/33, 67%; Supplementary Table S3). However, in our cohort, less than half (42%) of patients showed this pattern, suggesting a possible publication bias in the literature towards cases with severe WM involvement.

We observed a significant association of the supratentorial band‐like pattern with a higher TCS and with the presence of CSO calcification, regardless of patients' underlying genetic subtype, though more frequent in *PDGFB* and *MYORG* mutation carriers. Since all patients with band‐like involvement also showed CSO calcification, the latter could be an earlier stage of structural and biological changes reflecting different gene‐related disease mechanisms (including blood–brain barrier alteration, neurovascular unit dysfunction, pericytes and astrocytes alterations, differentiation pathway changes, ion transporter disfunction) leading to leukopathy.^2^, ^6^, ^29^ As the study design was cross‐sectional, longitudinal data are required to confirm the temporal relationship between calcification and leukopathy, and to define the evolution of the supratentorial WM band‐like pattern over time.

In our cohort, severe band‐like involvement was neither related to age (being found as early as the third decade), nor to the regional prevalence pattern (ie, anterior or diffuse), suggesting that this pattern is intrinsically related to PBC and not to ageing processes, although its pathogenesis remains to be clarified. Conversely, our analysis showed that the deep WM was always involved in subjects with band‐like involvement, suggesting that this area might be more vulnerable and that the alteration might subsequently spread to the contiguous periventricular and juxtacortical WM. Notably, even in the most severe cases, the U‐fibers appeared unaffected, with intertwined abnormal and normal‐appearing WM bands, rarely encountered in other leukodystrophies (except for rare cases of adult‐onset Alexander disease^30^); temporal and occipital WM was spared, suggesting a specific regional vulnerability. The isolated scattered supratentorial WM involvement did not differ in prevalence and severity from the vascular‐like changes observed in the control group as assessed by Fazekas score, supporting a shared small‐vessel disease etiology and no disease‐related increased risk for such changes.

As regards infratentorial WM alterations, cerebellar WM changes have largely been neglected in previous publications, although cerebellar calcifications are relatively common in PBC. The first report of cerebellar MRI changes in PBC^31^ was focused on the dentate nuclei, overlooking the concomitant changes of the MDB. In our cohort, 32% of patients showed cerebellar leukopathy, rated as severe in 75% of cases, with a potential association with some genetic subtypes. Regarding the pathogenesis of leukopathy, some authors have suggested that WM calcifications were the result of a long‐standing metabolic or inflammatory process leading to FLAIR/T2‐hyperintensity.^11^ As in our cohort the band‐like supratentorial involvement and cerebellar WM changes were always associated with high local calcification scores, while some patients had calcifications without WM changes, it is more likely that leukopathy was subsequent to the calcification process and not vice versa. Interestingly, band‐like supratentorial and cerebellar leukopathy mostly coexisted, showing a gradient from supratentorial to infratentorial involvement. In fact, cerebellar WM changes were always observed in patients with band‐like changes and in none with scattered or absent WM changes, while two‐thirds of patients with mild band‐like changes showed no cerebellar changes.

Regarding the cognitive correlates of imaging features, our study confirmed the association between supratentorial WM abnormalities in PBC and impaired cognitive performances, previously reported by Avrahami et al.^11^ In the absence of a consistent association between the severity and location of brain calcifications and the related clinical phenotype^4^, ^10^ the MRI assessment of WM might gain a relevant role. Notably, previous studies did not differentiate between a band‐like and a scattered vascular‐like pattern of WM changes. Considering our proposed classification, cognitive impairment was nearly twice as common in patients with a band‐like pattern compared with those with scattered WM changes, providing a possible structural underpinning for worse functional performance reported in some genotypes that are more frequently associated with WM changes.^4^, ^12^, ^13^, ^14^, ^15^, ^32^, ^33^ The band‐like supratentorial leukopathy could therefore become a potential imaging biomarker of cognitive impairment risk in PBC, based on its confirmation in larger cohorts. Conversely, PBC patients without band‐like involvement seem to present a vascular‐like brain ageing similar to the age‐matched control group and, with the exception of an outlier with a Fazekas score of 3, the expected relationship between higher Fazekas scores and lower cognitive performances. Taken together, these findings point to the importance of supratentorial WM radiological evaluation in PBC as (1) its involvement appears pivotal for cognitive performances, (2) it is related to local calcification processes in the band‐like phenotype, and (3) it is better characterized by MRI. However, other factors seem to play a role in cognitive impairment in PBC, such as WM alterations in cerebellar MDB, that have never been considered despite evidence supporting the role of the cerebellum in cognitive processes.^34^

Our results have also highlighted some associations between imaging features and cognitive domain deficits, that is, between band‐like supratentorial leukopathy and memory and between TCS and attention. These findings are in line with the main role of CSO and BG in these specific domains^35^ and will need confirmation by dedicated validation studies.

In the present study we also investigated the motor correlates of WM alterations. Cerebellar features and cerebellar atrophy are common in *MYORG* mutation carriers^32^, ^33^; in this genetic subgroup, cerebellar leukopathy was observed in 89% of our cases, being a major structural MRI counterpart of cerebellar dysfunction. Conversely, the relationship between non‐cerebellar motor impairment and supratentorial WM changes was less strong; in fact, WM changes correlated with a higher MDS‐UPDRS‐III score but not with the presence of other motor signs (eg, dystonia, tremor, etc.). This finding is in contrast with a previous study^11^ that did not show a correlation between WM involvement and the presence of parkinsonism. Likely, neurodegenerative processes locally associated with variable severity of calcification of BG and cerebellar nuclei might result in phenotypic heterogeneity.^2^, ^4^, ^9^, ^10^, ^35^, ^36^

Lastly, we also observed that psychiatric disturbances were present in nearly 90% of patients with band‐like supratentorial WM changes; psychiatric features are frequent in *PDGFB* mutations,^4^, ^5^, ^36^ and subcortical WM alterations might partially explain these symptoms, making WM changes a radiological marker with potential psychiatric implications. It has been hypothesized that WM changes in depressive disorder could be the result of several factors encompassing impaired blood–brain barrier function and inflammatory factors^37^ that have also been demonstrated in PBC animal models.

Even though the present study provides a detailed overview of WM changes in PBC, we acknowledge some limitations. First, the sample size of each PBC genetic subtype was relatively small, and the percentage of genetically undetermined patients (30% in our cohort) will likely decrease in the future, allowing re‐analysis of MRI data. In fact, routine application of whole genome sequencing could uncover intronic or structural variants not accounted for by NGS panels or whole exome sequencing.

Second, brain MRI was performed in a subset of cases of the Padua‐PBC cohort; our data might therefore not be representative of the whole clinical sample as the most severe patients could not take part in the study protocol.

Finally, this was a cross‐sectional study, and longitudinal data would certainly enrich our knowledge regarding pathogenesis, time course, and clinical relevance of WM involvement in PBC.

## 4 Conclusions

While CT remains the gold standard technique for detecting calcifications, our study provides evidence that brain MRI frequently highlights WM alterations in PBC, potentially contributing to explaining patients' motor, cognitive, and psychiatric heterogeneity. In particular, supratentorial band‐like and cerebellar leukopathy, but not scattered vascular‐like WM changes, may be a valuable imaging biomarker of clinical involvement.

## Author Roles

(1) Research Project: A. Conception, B. Organization, C. Data Acquisition, D. Data Analysis; (2) Statistical Analysis: A. Design, B. Execution, C. Review and Critique; (3) Manuscript Preparation: A. Writing of the First Draft, B. Review and Critique.G.L.: 1A, 1C, 1D, 3A.

G.B.: 1A, 1B, 1C, 1D, 2B, 3A.

M.Co.: 1C, 1D.

I.G.: 1B, 1C, 3A.

F.P.: 1C, 1D.

C.B.: 1C, 1D, 3B.

L.S.: 1C, 1D, 3B.

A.A.: 1C, 3B.

R.M.: 1A, 1C, 1D, 3B.

M.Ca.: 1A, 1C, 1D, 3B.

## Financial Disclosures and Conflicts of Interest

The authors have no conflicts of interest to disclose in relation to this research. No external financial support or funding was received for this work.

## Institutional Review Board Statement

The study was conducted in accordance with the Declaration of Helsinki and the Ethics Guidelines Committee from the home institution (Azienda Ospedale Università Padova).

## Informed Consent Statement

Informed consent was obtained from all subjects involved in the study for all study‐related procedures (including computed tomography scan, brain magnetic resonance imaging, and genetic testing), data collection, and publication.

## Supporting information


**Table S1.** Comparison between primary brain calcification (PBC) patients enrolled and not enrolled in the study. SD: standard deviation.
**Table S2.** Articles reporting on supratentorial and infratentorial white matter changes in primary brain calcification. Leukopathy was assessed by consensus of one senior neuroradiologist (R.M.) and one senior radiology resident (I.G.) based on the figures of the articles. M, male; F, female; PDGFB, platelet‐derived growth factor subunit B; XPR1, xenotropic and polytropic retrovirus receptor 1; SLC20A2, solute carrier family 20 member 2; n/a, not available; BG, axial section at the level of the basal ganglia; BGc, coronal section at the level of the basal ganglia; *, ischemic lesion in the right occipital lobe, cerebral amyloid angiopathy.
**Table S3.** Summary of the features of supratentorial and infratentorial white matter changes in primary brain calcification patients from the literature. PDGFB, platelet‐derived growth factor subunit B; n/a, not available; XPR1, xenotropic and polytropic retrovirus receptor 1; SLC20A2, solute carrier family 20 member 2.

## Data Availability

The data that support the findings of this study are available from the corresponding author upon reasonable request.
