# Supplementary material for: White Matter Matters: A Magnetic Resonance Imaging Study with Clinical Correlates in Primary Brain Calcification
Source: Mov Disord. 2026 Feb 21;41(6):1555–65. doi: 10.1002/mds.70249 (PMC13307233; doi:10.1002/mds.70249)
Supplement: Supplementary file 1 — Table S1. Comparison between primary brain calcification (PBC) patients enrolled and not enrolled in the study. SD: standard deviation. Table S2. Articles reporting on supratentorial and infratentorial white matter changes in primary brain calcification. Leukopathy was assessed by consensus of one senior neuroradiologist (R.M.) and one senior radiology resident (I.G.) based on the figures of the articles. M, male; F, female; PDGFB, platelet‐derived growth factor subunit B; XPR1, xenotropic and polytropic retrovirus receptor 1; SLC20A2, solute carrier family 20 member 2; n/a, not available; BG, axial section at the level of the basal ganglia; BGc, coronal section at the level of the basal ganglia; *, ischemic lesion in the right occipital lobe, cerebral amyloid angiopathy. Table S3. Summary of the features of supratentorial and infratentorial white matter changes in primary brain calcification patients from the literature. PDGFB, platelet‐derived growth factor subunit B; n/a, not available; XPR1, xenotropic and polytropic retrovirus receptor 1; SLC20A2, solute carrier family 20 member 2. [file MDS-41-1555-s001.docx]

**White matter matters: an MRI study with clinical correlates in Primary Brain Calcification**

**Supplementary material**

|  | **N** | **%** | **p vs included subjects** |
| --- | --- | --- | --- |
| Females | 24 | 64.9% | 0.2 |
| Known genetic cause | 12 | 32.5% | **0.02** |
| Age (SD) | 62.5 (15.6) |  | **0.048** |
| Disease duration (SD) | 8.9 (5.7) |  | 0.7 |
| TCS (SD) | 17.05 (12) |  | 0.4 |
| Asymptomatic subj. | 8 | 21.6% | 0.9 |
| Motor symptoms | 26 | 70.3% | 0.2 |
| Parkinsonism | 17 | 46% | 0.53 |
| Cerebellar signs | 6 | 16% | 0.57 |
| NPSI | 23 | 62% | 0.3 |
| Cognitive decline | 20 | 54% | 0.2 |

**Supplementary Table 1.** Comparison between PBC patients enrolled and not enrolled in the study. SD: standard deviation

| **First author** | **Publication year** | **Gender** | **Age (y)** | **Genetics** | **Centrum semiovale (T2/FLAIR)** | | | | **Medullary corpora** |
| --- | --- | --- | --- | --- | --- | --- | --- | --- | --- |
|  |  |  |  |  | **Prevalence** | **Severity** | **Pattern** | **Region** | **Grading** |
| Scotti | 1985 | F | 50 | n/a | diffuse | severe | band-like | Deep/periventricular with intermediate sparing | no |
| Avrahami | 1994 | M | 41 | n/a |  |  | scattered (BG) |  | n/a |
| Ogi | 2002 | F | 61 | n/a | diffuse | severe | band-like | Deep/periventricular/iuxtacortical | n/a |
|  |  | M | 63 | n/a | diffuse | severe | band-like | Deep/periventricular/iuxtacortical | n/a |
|  |  | M | 66 | n/a | diffuse | severe | band-like (BG) | Deep/periventricular | severe |
| de Albuquerque Cavalcanti-Mendes Gde | 2009 | M | 51 | n/a | diffuse | severe | band-like (BG) | Deep/periventricular/iuxtacortical | n/a |
| Kozic | 2009 | M | 64 | n/a | diffuse | severe | band-like (BG) | Deep/periventricular/iuxtacortical | severe |
| Sentimentale | 2010 | F | 58 | n/a | diffuse | severe | band-like (BGc) | Deep/periventricular with intermediate sparing | n/a |
| Govindarajan | 2013 | M | 30 | n/a | diffuse | severe | band-like | Deep/periventricular/iuxtacortical | mild |
| Nicolas | 2014 | F | 77 | PDGFB | diffuse | severe | band-like | Deep/periventricular with intermediate sparing | no |
| Lo Bono | 2015 | F | 43 | n/a |  |  | scattered cBG |  | n/a |
| Sahin | 2015 | M | 74 | n/a | diffuse | severe | band-like | Deep/periventricular /iuxtacortical | severe |
|  |  | F | 37 | n/a |  |  | no (BG) |  | n/a |
| Keogh | 2015 | F | 60 | PDGFB |  |  | scattered |  | no |
|  |  | F | 20 | PDGFB |  |  | no |  | n/a |
| Anheim | 2016 | M | 41 | XPR1 | diffuse | mild | band-like (BG) | periventricular | n/a |
| Biancheri | 2016 | F | 5 | PDGFB | anterior | mild | band-like | periventricular | n/a |
|  |  | F | 22 | PDGFB | anterior | mild | band-like (cBG) | Deep/periventricular/iuxtacortical | n/a |
|  |  | F | 44 | PDGFB |  |  | scattered |  | n/a |
|  |  | F | 72 | PDGFB |  |  | scattered |  | n/a |
|  |  | M | 42 | PDGFB | diffuse | mild | band-like | Deep/periventricular | n/a |
| Matsusaka | 2018 | M | 74 | n/a | diffuse | severe | band-like | Deep/periventricular | n/a |
| Sakai | 2022 | M | 70 | SLC20A2 | diffuse | severe | band-like | Deep/periventricular/iuxtacortical | n/a |
| Shen J | 2022 | F | 24 | PDGFB |  |  | scattered |  | n/a |
|  |  | F | >70 | PDGFB | diffuse | severe | band-like | deep/periventricular | n/a |
| Al Ali | 2023 | F | 51 | PDGFRB | diffuse | severe | band-like | Deep/periventricular with intermediate sparing | mild |
| Shahid | 2023 | F | 42 | n/a | diffuse | severe | band-like | deep/periventricular/iuxtacortical | severe |
| Farahmand | 2024 | M | 66 | PDGFB | diffuse | severe | band-like | deep/periventricular/iuxtacortical | n/a |
|  |  | M | 45 | PDGFB |  |  | no |  | n/a |
|  |  | F | 87 | PDGFB | diffuse | severe | band-like | deep/periventricular/iuxtacortical | n/a |
|  |  | F | 27 | PDGFB |  |  | scattered |  | n/a |
|  |  | F | 53 | PDGFB |  |  | scattered |  | n/a |
|  |  | F | 72 | PDGFB | diffuse | severe | band-like (*) | Deep/periventricular with intermediate sparing | n/a |

**Supplementary Table 2**. Articles reporting on supratentorial and infratentorial white matter changes in Primary Brain Calcification. Leukopathy was assessed by a consensus of one senior neuroradiologist (R.M.) and one senior radiology resident (I.G.) based on the figures of the articles. M: male; F: female; PDGFB: Platelet-derived growth factor subunit B; XPR1: Xenotropic and polytropic retrovirus receptor 1; SLC20A2: Solute carrier family 20 member 2; n/a: not available; BG: axial section at the level of the basal ganglia; BGc: coronal section at the level of the basal ganglia; *: ischemic lesion in the right occipital lobe, cerebral amyloid angiopathy

| **Feature** |  | **N** | **Notes** |
| --- | --- | --- | --- |
| *All patients* |  | 33 | mean age±standard deviation = 61±15,5 years |
| **Gender** | female | 20 |  |
|  | male | 13 |  |
| **Genetics** | PDGFB | 17 |  |
|  | n/a | 14 |  |
|  | XPR1 | 1 |  |
|  | SLC20A2 | 1 |  |
| **Medullary corpora** | severe | 4 | all presenting with band-like diffuse and severe supratentorial leukopathy |
|  | mild | 2 | all presenting with band-like diffuse and severe supratentorial leukopathy |
|  | no | 3 |  |
|  | n/a | 24 |  |
| **Centrum semiovale** | band-like | 22 | diffuse-severe = 18  diffuse-mild = 2  anterior-mild = 2 |
|  | scattered | 8 |  |
|  | no | 3 |  |

**Supplementary Table 3**. Summary of the features of supratentorial and infratentorial white matter changes in Primary Brain Calcification patients from the literature. PDGFB: Platelet-derived growth factor subunit B; n/a: not available XPR1: Xenotropic and polytropic retrovirus receptor 1; SLC20A2: Solute carrier family 20 member 2.
